# Desultory Notes of a Medical Traveller

**Published:** 1841-02

**Authors:** 


### WESTERN JOURNAL

OF

### MEDICINE AND SURGERY.

FEBRUARY, 1841.

Art. I.—*Desultory Notes of a Medical Traveller.* In a letter to one of the Editors, dated Staunton, Virginia, July 7th, 1840.

On Saturday, the 4th inst., I found myself among a large assembly of people that had collected from various points to witness the commencement of the University at Charlottesville, an institution of which Virginians may justly feel proud. Among the graduates were eight who received the honors of our profession, at least so far as the degree of this institution could confer them; for on conversing with some of them, I

soon discovered they did not consider their education completed, as, notwithstanding the rigorous examinations through which they had passed, all expected to attend some other school for a degree—the University of Pennsylvania appeared to be the favorite with most of them, though State pride will induce others to go to the new school at Richmond;—Louisville presents the greatest attractions for all who expect to settle in the great Valley of the Mississippi.

You should have received a more extended account of this institution, and of the pleasant town near which it is situated, but my visit was very short, and unfortunately on a gala day, when all were intent upon the approaching festival and the ceremonies of commencement. Every thing appeared in holiday garb, which you know is not very favorable for critical scrutiny; my impressions were very agreeable, and incline me to accord to the institution a very high rank,—one which, perhaps, even exceeds the most sanguine anticipations of its patriotic founder. Of the Professors in the medical department I can say nothing, not having had the good fortune to meet any of them. They appear to stand high, but their practice must be limited, as this is a remarkably healthy region, not densely inhabited, the town small and containing other physicians. These circumstances, in connexion with the small size of the class, and the want of a hospital, must render it any thing but a desirable residence for one ambitious for distinction in the profession.

Of the buildings, I may say that they are commensurate with the expanded views of their projector, who, from the heights of Monticello, looked upon the beautiful undulating plain at the base of the Blue Ridge, observed its capabilities, and, prompted, no doubt, by the most benevolent motives, determined to erect an institution worthy of his glorious state. The arrangement is rather diffuse, but the effect is good when observed from certain points. The plan is a very expensive one, and every thing has been constructed in the best manner. The lecture rooms are in a large building, the college proper, that also contains a magnificent rotunda, the depository of an extensive and valuable library. The upper gallery of this rotunda is appropriated to objects of natural history, where I expected to have found complete suites of the Virginia naiades, and at least a collection of specimens illustrating the interesting geology of this great state: neither the one nor the other is there—excepting a suite of the tertiary fossils of the tide water marls; though they have a very fine mineralogical cabinet from all other parts of the world.

The road from Charlottesville passes through a fine rolling country, to the foot of the Blue Ridge that is crossed at Rock-Fish Gap, where the traveller is presented with a glorious prospect to the south and east. Here, we bid adieu to the "Old Dominion," and leave behind us the metamorphic rocks with their auriferous quartz and dykes of trap, a series that are exciting very great interest among geologists, some of whom have supposed that the micaceous schists and epidotic rocks of this region are altered shales, so modified by the irruptions of trap as to have lost all their external distinctive characters. On the western declivity of the mountain, the road is rough in consequence of the strata dipping to the south-east, but no opportunity was presented for tracing their connexion with the limestones of the "Valley of Virginia." These rocks appear identical with those of the Cumberland valley of Pennsylvania—they are all upturned at a considerable angle, and in some places, perhaps overturned, especially near the Blue Ridge. It is in such a situation that the celebrated Weir's cave is found, and it is very probable that the confusion of the strata in the overthrow may have aided in the formation of this extraordinary and most beautiful cavern, the wonderful productions of which I shall not attempt to describe, as nothing but the pen accustomed to voyage in fairy land could possibly do justice to the surpassing beauty of the scene. The curious figures and memnonian sounds are alluded to in the following lines:

In yonder grot
Where forms fantastic hang on either hand,
Where jewels glisten like the golden sand,
And every tint that glows in western skies
Is there reflected, as from Argus' eyes,
While the mute gazer stands, or gropes along
The pipes of crystal hail him with their song—
For there — so far within the terrene world
The rocks are struck, and music is unfurled.

ANONYMOUS.

But leaving the cave and its poets, and returning to sober prose, I must advert to some of the general features of this "Valley." Bounded by the Blue Ridge and North mountain, it embraces a large extent of rolling limestone land, some of which is very fertile, and in which rise some of the principal rivers in the state. I am now nearly midway between the mountains, in an elevated region on the head waters of the Shenandoah. This town has been selected for its healthy and central situation as the site of several public charities, among which are the Lunatic Asylum, and schools for the blind, and for deaf mutes The latter are still in their infancy, though a fine building is in progress for the mutes; the corner stone is to be laid with due ceremony to-morrow. Great additions are also making to the Lunatic Asylum, already a large edifice, but insufficient for the wants of the state. I

was gratified to find that the moral treatment was chiefly relied on in the management of this institution; at least, this appears the leading principle of action, and the happiest results are already flowing from it to this unfortunate class of sufferers, whose treatment upon the old one of cruel confinement, and even chains, has so often excited our sympathies. Dr. Stribling's judicious management, and the arrangements he has suggested for the comfort, entertainment and occupation of these unfortunate beings, are producing one among the numerous results of the reformation begun within half a century by the good Pinel. I have the annual report of the attending physician before me, but cannot at present notice it further than to extract some statistics which will prove interesting and useful:

| | Males. | Females. | Total. |
|---|---|---|---|
| Old patients at the beginning of the year, - | 37 | 35 | 72 |
| Admitted since, - - - - - | 19 | 13 | 32 |
| Whole number, - - - - | 56 | 48 | 104 |

| | Males. | Females. | Total. |
|---|---|---|---|
| Discharged during the same period, - | 16 | 8 | 24 |
| Died " " " - - | 3 | 4 | 7 |
| Eloped " " " - - | 2 | 0 | 2 |
| | | | 33 |
| Leaving in the Institution, - - - - - - | | | 71 |

*Civil Condition.*

| | Males. | Females. | Total. |
|---|---|---|---|
| Married, - - - - - - | 11 | 18 | 29 |
| Single, - - - - - - | 38 | 21 | 59 |
| Widowed, - - - - - - | 3 | 6 | 9 |
| Unknown, - - - - - - | 4 | 3 | 7 |
| | | | 104 |

*Duration of the Disease up to the present time, discharge or death.*

| | |
|---|---|
| Less than one year, | 20 |
| From one to five years, | 30 |
| From five to ten years, | 19 |
| From ten to twenty years, | 27 |
| From twenty to thirty years, | 1 |
| | 104 |

*Respective Ages of Patients.*

| | |
|---|---|
| Under 20 years, | 8 |
| Between 20 and 30 years, | 25 |
| " 30 " 40 " | 33 |
| " 40 " 50 " | 24 |
| " 50 " 60 " | 10 |
| " 60 " 70 " | 3 |
| " 70 " 80 " | 1 |
| | 104 |

*Form of the Disease.*

| | |
|---|---|
| Monomania, | 11 |
| Mania, | 48 |
| Melancholia, | 28 |
| Hypochondriasis, | 7 |
| Dementia, | 9 |
| Idiocy, | 1 |
| | 104 |

*Causes in the thirty-five last Received.*

| | | | |
|---|---|---|---|
| *Moral Causes,* | 14 | *Physical Causes,* | 19 |
| Domestic affliction | 4 | Intemperance, | 4 |
| Ungoverned temper, | 1 | Ill health, | 8 |
| Family troubles, | 4 | Puerperal, | 3 |
| Disappointed affection, | 1 | Epilepsy, | 3 |
| Reverse in fortune, | 2 | Mastubation, | 1 |
| Religious excitement, | 2 | Unknown, | 2 |
| Intense study, | 1 | | |

| | Males. | Females. | Total. |
|---|---|---|---|
| Of recent Cases, - - - - - | 13 | 7 | 20 |
| Cured, - - - - - - - | 10 | 6 | |
| Much improved, - - - - - - | 1 | 1 | |
| Eloped, - - - - - - - | 1 | 0 | |
| Died, - - - - - - - - | 1 | 0 | |

From these tables it will appear, that of recent cases (less than a year) from 80 to 90 per cent have been cured, and Dr. Stribling states, that of the whole number admitted since the improved system has been adopted, 43-100 have recovered. These are truly flattering results, which speak volumes in favor of the moral treatment, or a happy combination of exercise, occupation, amusement, freedom and restraint, with due attention to comfort.

But I must conclude this long letter, hoping to find something of interest to communicate to you from the western part of the State. J. A. W.

